# The Efficacy of Commercial Surface Sanitizers against Norovirus on Formica Surfaces with and without Inclusion of a Wiping Step

**DOI:** 10.1128/aem.00807-22

**Published:** 2022-08-25

**Authors:** Jeremy Faircloth, Rebecca M. Goulter, Clyde S. Manuel, James W. Arbogast, Blanca Escudero-Abarca, Lee-Ann Jaykus

**Affiliations:** a Department of Food, Bioprocessing and Nutrition Sciences, North Carolina State University, Raleigh, North Carolina, USA; b GOJO Industries, Inc., Akron, Ohio, USA; Centers for Disease Control and Prevention

**Keywords:** human norovirus, Tulane virus, sanitize, disinfect, wiping

## Abstract

Commonly used surface sanitizers often lack activity against human noroviruses (hNoVs). The impact of inactivation versus removal when these products are applied via wiping is poorly characterized. The purpose of this work was to assess the anti-hNoV efficacy of various surface sanitizer chemistries, as applied to a laminate material commonly used for restaurant tabletops, using standard surface assays (ASTM E1053-11) and a newly developed wiping protocol. Four commercially available products with different active ingredient(s) (i.e., ethanol [EtOH], acid + anionic surfactant [AAS], quaternary ammonium compound [QAC], and sodium hypochlorite [NaOCl]) and a water control were evaluated against hNoV GII.4 Sydney, hNoV GI.6, and the cultivable surrogate Tulane virus (TuV). Virus concentration was evaluated using RNase-reverse transcriptase (RT)-quantitative PCR (qPCR) (hNoV) and infectivity assay (TuV). Only the EtOH-based product significantly reduced virus concentration (>3.5 log_10_ reduction [LR]) by surface assay, with all other products producing ≤0.5 LR. The inclusion of a wiping step enhanced the efficacy of all products, producing complete virus elimination for the EtOH-based product and 1.6 to 3.8 LR for the other chemistries. For hNoVs, no detectable residual virus could be recovered from paper towels used to wipe the EtOH-based product, while high concentrations of virus could be recovered from the used paper towel and the wiped coupon (1.5 to 2.5 log_10_ lower genome equivalent copies [GEC] compared to control) for the QAC- and AAS-based products and for water. These results illustrate the variability in anti-hNoV activity of representative surface sanitizers and highlights the value of wiping, the efficacy of which appears to be driven by a combination of virus inactivation and removal.

**IMPORTANCE** Human noroviruses (hNoVs) are the leading cause of acute gastroenteritis and food-borne disease worldwide. Noroviruses are difficult to inactivate, being recalcitrant to sanitizers and disinfectants commonly used by the retail food sector. This comparative study demonstrates the variability in anti-hNoV activity of representative surface sanitizers, even those allowed to make label claims based on the cultivable surrogate, feline calicivirus (FCV). It also highlights the importance of wiping in the process of sanitization, which significantly improves product efficacy through the action of physical removal of surface microbes. There is a need for more and better product formulations with demonstrated efficacy against hNoVs, which will likely necessitate the use of alternative cultivable surrogates, such as Tulane virus (TuV). These findings help food safety professionals make informed decisions on sanitizing product selection and application methods in order to reduce the risk of hNoV contamination and transmission in their facilities.

## INTRODUCTION

Human noroviruses (hNoVs) are the most common cause of diarrheal illness around the world, with 684 million cases occurring annually (1). Recent estimates of the burden of hNoV illness suggest that this virus group is responsible for over 200,000 deaths each year (1) with an annual global economic impact of $4.2 billion in direct health care costs and $60.3 billion in societal costs (2). In the United States, hNoVs are the most common cause of food-borne illness, with an estimated 5.5 million cases annually (3). Immunity to hNoVs is short-lived (4), and no licensed vaccines that provide long-term protection are currently available (5).

Human norovirus transmission readily occurs via multiple routes, including directly through person-to-person contact and indirectly by environmental contamination on surfaces (fomites) or through food-borne or waterborne routes (6). The viruses are transmitted readily and infect humans efficiently, due to their low infectious dose (estimated to be 18 to 1,000 viral particles) (7, 8), high concentrations in the feces of infected individuals (10^5^ to 10^11^ viral RNA copies/g) (9), and high degree of environmental persistence (on surfaces, they maintain infectivity for weeks) (10). Additionally, given their nonenveloped structure, hNoVs are highly resistant to a broad range of sanitizers and disinfectants (11, 12). In fact, the U.S. Centers for Disease Control and Prevention (CDC) recommends solutions of 1,000 to 5,000 ppm of sodium hypochlorite to inactivate hNoVs during a suspected contamination event (13). The use of such high-sodium hypochlorite concentrations is not tenable in many venues due to its corrosivity and/or potential to stain many hard and soft surface materials, a strong polarizing odor, potential health risks and hazards to the user, and susceptibility to reduced efficacy in the presence of organic material (14). In addition, the maximum allowable concentration of free chlorine for sodium hypochlorite-based surface sanitizers is 200 ppm when used on food contact surfaces (FCSs) (15). Any chlorine-based product containing higher concentrations requires a rinse step with potable water after application on FCSs (15).

Quaternary ammonium compounds (QACs) are a class of active ingredients frequently found in surface sanitizers intended for use on FCSs. Their use in this capacity does not require a potable water rinse after application if the use solution contains 150 to 400 ppm QAC (15). While QAC-containing surface sanitizers are popular due to their relatively low cost and broad bacterial efficacy, they have been shown to be relatively ineffective at inactivating nonenveloped viruses, including hNoVs (11, 12, 16, 17). Considering the limitations of chlorine- and QAC-based products, alternative FCS sanitizers with enhanced hNoV efficacy and more favorable safety and material compatibility profiles are needed to mitigate environmental and food-borne transmission of this pathogen.

Given the historical difficulties of cultivating hNoVs, studies investigating the efficacy of surface sanitizers and disinfectants have traditionally relied on the use of cultivable surrogate viruses (e.g., feline calicivirus [FCV], murine norovirus [MNV], among others) or PCR-based assays (reverse transcriptase [RT]-quantitative PCR [qPCR]), often preceded by an RNase pretreatment to destroy partially encapsidated or naked RNA that is not infectious (11, 12, 16, 18–20). Both approaches have limitations. Surrogate viruses have been shown to be highly variable in how they respond to various antimicrobial chemistries compared to hNoVs (11, 12, 20), and RT-qPCR, even with an RNase pretreatment, does not provide an exact measure of infectious virus (21). Currently, the U.S. Environmental Protection Agency (EPA) recognizes FCV as the preferred surrogate virus, the data for which provide the basis for making anti-hNoV label claims for EPA-registered products (22). However, FCV is more sensitive to pH extremes and chlorine than hNoVs (12), meaning that there is high potential for misleading performance claims when using FCV to approximate hNoV inactivation characteristics. The recently identified Tulane virus (TuV) (23), a calicivirus first discovered in rhesus macaques, is considered to be a better surrogate given its structural similarity and susceptibility to antimicrobial chemistries compared to hNoVs (12). To our knowledge, comparative studies investigating the efficacy of surface sanitizers and disinfectants against both TuV and hNoVs using the same study design are scarce. Such studies are needed to assess the true efficacy of these products against the clinically relevant hNoVs.

Many surface sanitizers and disinfectants are utilized via the action of wiping with cloth or paper towels. When product implementation is done by wiping after wetting the surface, the efficacy of these products will be mediated by the combined effects of virus inactivation and physical removal. Indeed, the application method of a surface sanitizer or a disinfectant has been shown to significantly affect antiviral efficacy. For instance, Gibson et al. (24) demonstrated effective removal of hNoV surrogates from stainless steel and laminate surfaces by wiping with different textiles that were moistened using water alone. Another study showed enhanced efficacy of chemically impregnated towelettes against the hNoV surrogate MNV, presumably due to facilitating virus removal from a surface (16). While these previous studies investigated surrogate viruses, to our knowledge, studies investigating the importance of the wiping step in overall reduction in hNoV concentration during surface sanitization and disinfection have not yet been performed. Additionally, studies comparing the performance of commercially available FCS sanitizers against hNoVs on typical tabletop surface materials used by the retail food sector (i.e., the laminate material sometimes referred to by the trade name Formica) have not yet been done.

One reason for the absence of previous studies is that evaluating performance of a sanitizer or disinfectant in conjunction with a wiping step can be difficult to perform in a controlled and consistent manner. Two standardized wiping methods have been used in the past to evaluate antiviral efficacy, i.e., the Wiperator machine (ASTM E2967-15) and the 4-field test (EN 16615), but they each have limitations. The 4-field test uses a manual wiping method with a unitary weight, with wiping performed across a large surface using two passes of horizontal motion. The Wiperator is an automated mechanical method that applies a repeated orbital wiping motion to a small removable coupon, theoretically allowing for more consistent wiping along with easier, more efficient residual virus recovery. However, the Wiperator method does not easily facilitate spray application, and the wiping action may not be representative of real-use conditions due to an unrealistically high wiping pressure, as a result of using a low contact surface area for the wipe relative to the force applied by the 150-g mass (25). While the 4-field test is generally considered more reflective of real-world conditions, it lacks the consistency and ease of sample recovery experienced using the automated method with removable coupons (25).

In this study, we used controlled antiviral surface assays to assess the relative anti-hNoV efficacy of four commercially available surface sanitizer chemistries, as applied to a commonly used restaurant tabletop material. Additionally, we developed an automated wiping platform that facilitates spray application of products and used this method to perform controlled experiments to assess the added benefit of incorporating a paper towel wiping step in the sanitization of surfaces.

## RESULTS

**FIG 1:**
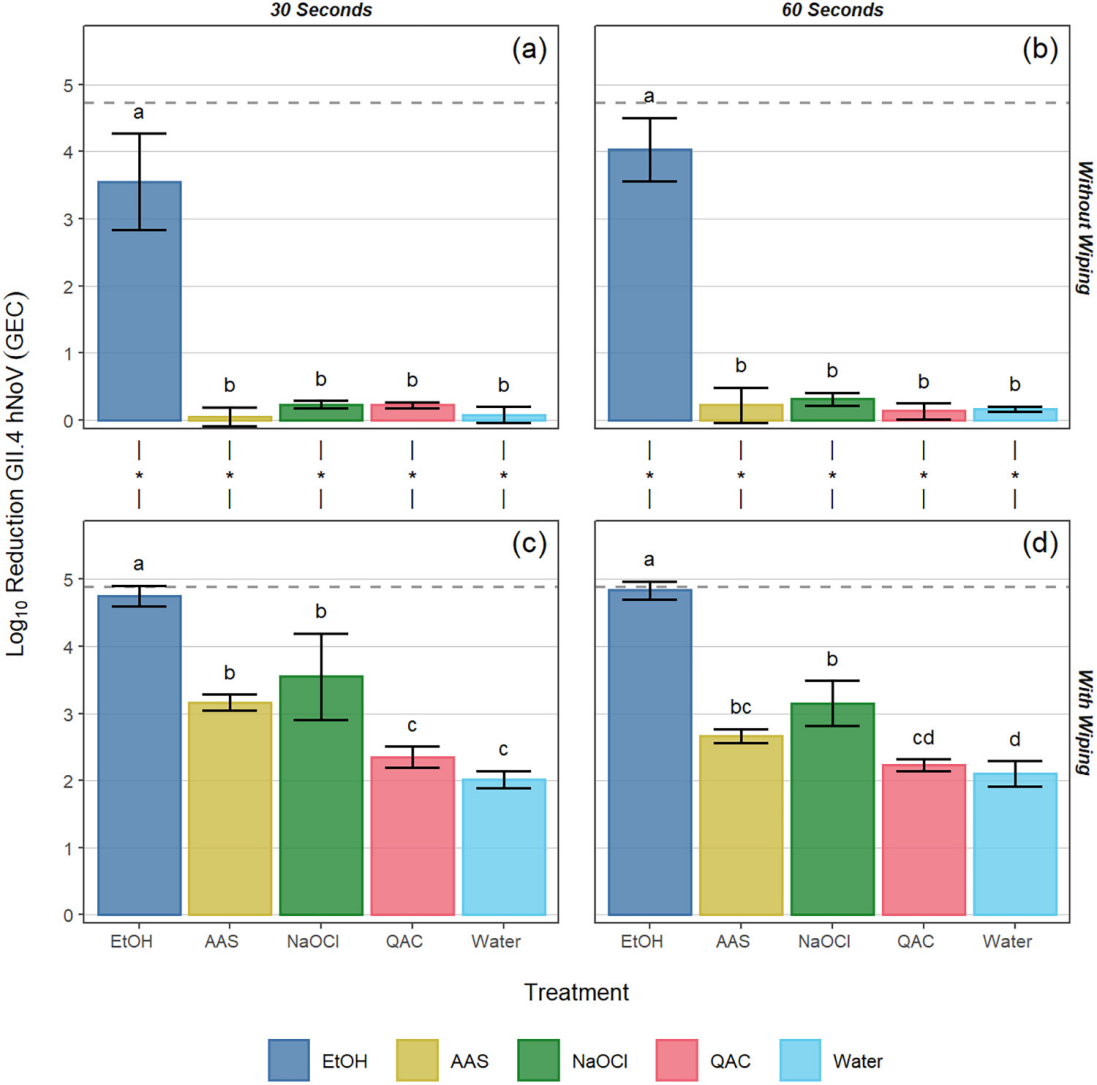
Log_10_ reduction of GII.4 human norovirus (hNoV) genome equivalent copies (GECs) by various sanitizing products and contact times (30 and 60 s) on Formica coupons without (a, b) and with (c, d) a wiping step with paper towel. The dotted line for each panel represents the limit of detection of the assay. Different letters within a panel represent significant statistical differences (*P < *0.05) in the log_10_ reduction of GII.4 hNoV GEC when comparing products within that panel. Asterisks used in the margin above panels c and d indicate situations in which a statistically significant (*P < *0.05) increase in log_10_ reduction of GII.4 hNoV GEC occurred as a result of incorporating a wiping step into the process. Error bars represent the standard deviation. EtOH, ethanol; AAS, acid + anionic surfactant; NaOCl, sodium hypochlorite; QAC, quaternary ammonium compound.

**FIG 2:**
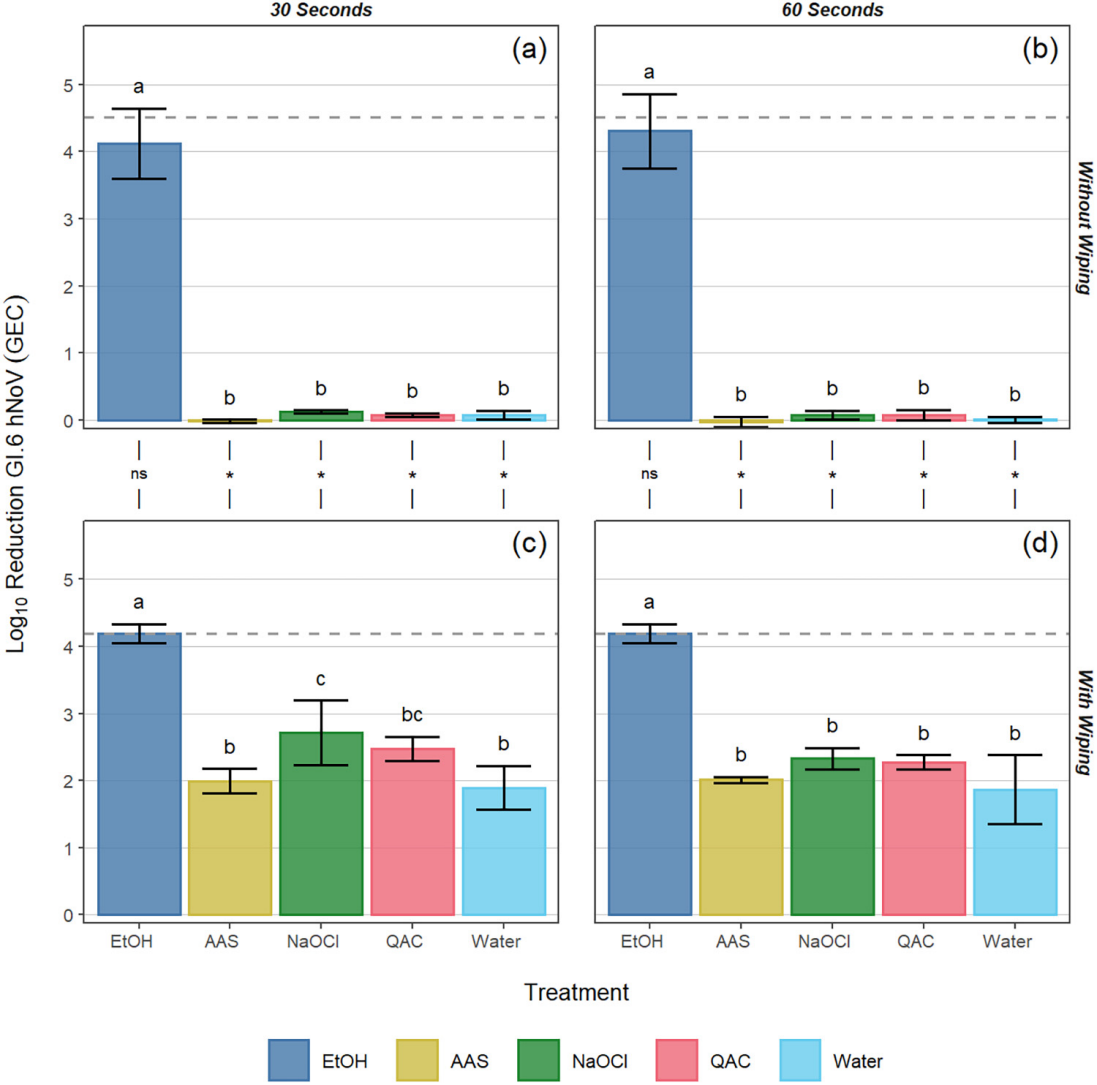
Log_10_ reduction of GI.6 hNoV GEC by various sanitizing products and contact times (30 and 60 s) on Formica coupons without (a, b) and with (c, d) a wiping step with paper towel. The dotted line for each panel represents the limit of detection of the assay. Different letters within a panel represent significant statistical differences (*P* < 0.05) in the log_10_ reduction of GI.6 hNoV GEC when comparing products within that panel. Asterisks used in the margin above panels c and d indicate situations in which a statistically significant (*P* < 0.05) increase in log_10_ reduction of GI.6 hNoV GEC occurred as a result of incorporating a wiping step into the process. Error bars represent the standard deviation. EtOH, ethanol; AAS, acid + anionic surfactant; NaOCl, sodium hypochlorite; QAC, quaternary ammonium compound.

**FIG 3:**
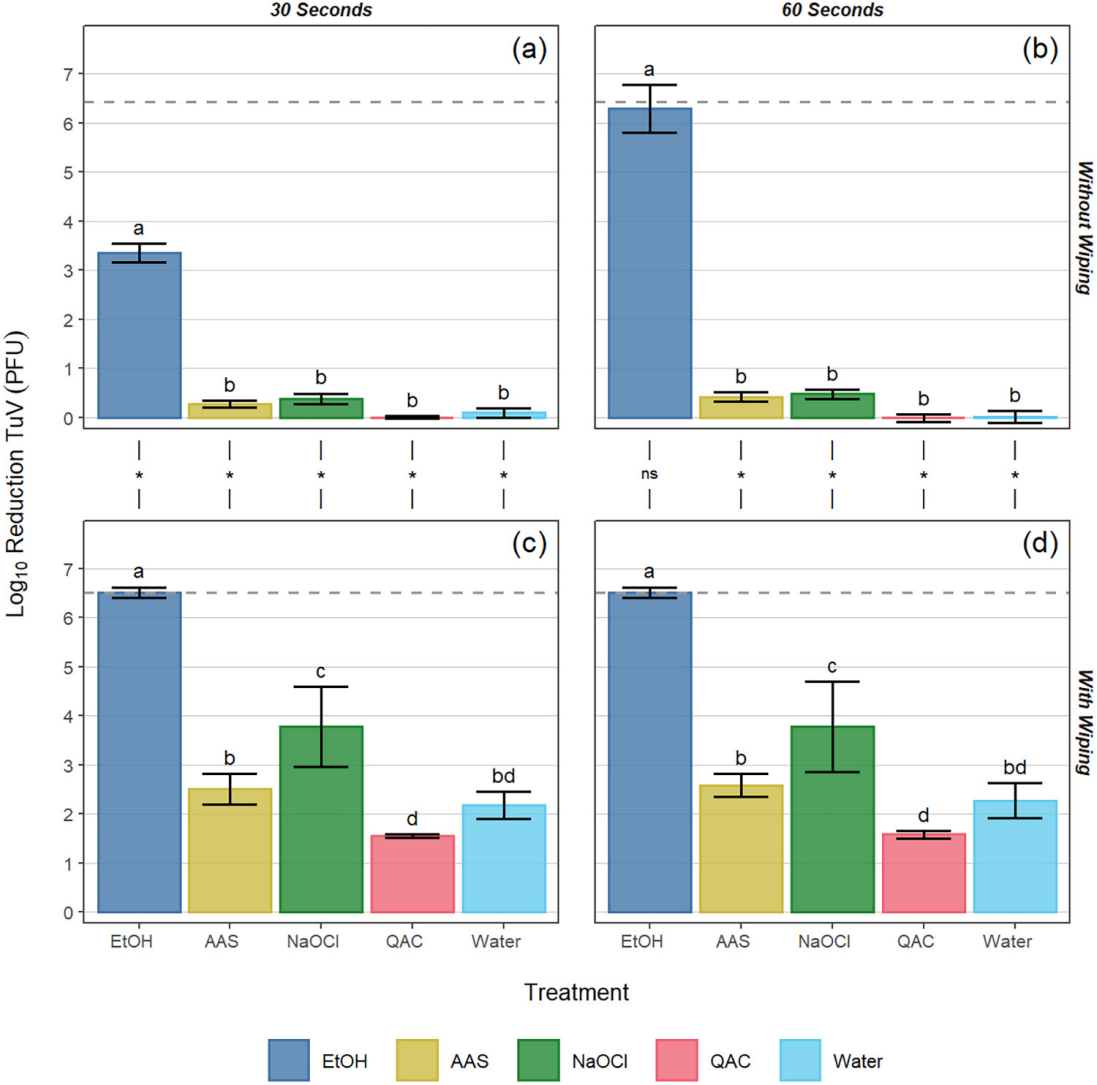
Log_10_ reduction of Tulane virus (TuV) PFU by various sanitizing products and contact times (30 and 60 s) on Formica coupons without (a, b) and with (c, d) a wiping step with paper towel. The dotted line for each panel represents the limit of detection of the assay. Different letters within a panel represent significant statistical differences (*P* < 0.05) in the log_10_ reduction of TuV PFU when comparing products within that panel. Asterisks used in the margin above panels c and d indicate situations where a statistically significant (*P* < 0.05) increase in log_10_ reduction of TuV PFU occurred as a result of incorporating a wiping step into the process. Error bars represent the standard deviation. EtOH, ethanol; AAS, acid + anionic surfactant; NaOCl, sodium hypochlorite; QAC, quaternary ammonium compound.

In standard ASTM surface assays (no wiping step) following a 30-s contact time with the EtOH-based product, log_10_ reductions of 3.6 ± 0.7, 4.1 ± 0.5, and 3.4 ± 0.2 were observed for GII.4, GI.6, and TuV, respectively. Treatment with all other products at the 30-s contact time resulted in statistically significantly lower reductions in viral titer (≤0.5 log_10_) compared to the EtOH-based product (*P* < 0.05; Fig. 1a, 2a and 3a). When the contact time for the EtOH-based product was extended to 60 s, log_10_ reductions of 4.0 ± 0.5, 4.3 ± 0.6, and 6.3 ± 0.5 were observed for GII.4, GI.6, and TuV, respectively (Fig. 1b, 2b, and 3b). Similar to the 30-s exposure time, the other formulated or diluted products included in this study produced ≤0.5 log_10_ reduction at 60 s, significantly lower than those observed for the EtOH-based product (*P* < 0.05; Fig. 1b, 2b, and 3b).

As expected, the inclusion of a wiping step provided greater log_10_ reductions in virus concentration for all products tested against all viruses (*P* < 0.05), with the only exceptions being cases in which the EtOH-based product was already at or near the limit of detection (LOD) when tested without a wiping step. For the EtOH-based product, the inclusion of the wiping step resulted in log_10_ reductions reaching the assay limit of detection for all contact times against GI.6 and TuV (LOD of 4.2 and 6.5 log_10_ reduction for GI.6 and TuV, respectively), whereas log_10_ genome equivalent copy (GEC) reductions of 4.8 ± 0.2 and 4.8 ± 0.1 were observed for GII.4 at 30 and 60 s, respectively (LOD of 4.9 log_10_ reduction for GII.4). These log_10_ reductions were significantly higher than those for the remaining chemistries (*P* < 0.05; Fig. 1c and d, 2c and d, and 3c and d).

For GII.4 wiping assays following a 30-s contact time, log_10_ hNoV GEC reductions ranging from 2.4 to 3.6 were observed for the AAS-, NaOCl-, and QAC-based products, while the water control produced a 2.0 log_10_ hNoV GEC reduction (Fig. 1c). For the 60-s contact time, log_10_ hNoV GEC reductions ranging from 2.2 to 3.2 were observed for the AAS-, NaOCl-, and QAC-based products, while the water control produced a 2.1 log_10_ hNoV GEC reduction. No statistically significant difference was observed by analysis of variance (ANOVA) when comparing contact times against GII.4 hNoV (*P* = 0.098; Fig. 1c and d). Compared to the water control at 30 s, significantly higher reductions were observed for the NaOCl (*P* < 0.001) and AAS-based (*P* = 0.003) products, along with the EtOH-based (*P* < 0.001) product, with no differences observed between the QAC-based product and the water control (*P* = 0.715; Fig. 1c). Similar trends were observed in the GI.6 wiping assays, for which log_10_ GEC reductions ranged from 2.0 to 2.7 for the AAS-, NaOCl-, and QAC-based products, again with no overall significant differences observed between contact times (*P* = 0.231). In most cases, wiping with these sanitizers produced GI.6 log_10_ GEC reductions that were not significantly different from that of the water control (*P* > 0.05), with the only exception being NaOCl at 30 s (*P* = 0.009; Fig. 2c and d). For TuV wiping assays, the NaOCl-based product outperformed the AAS-based (30 s, *P* < 0.001; 60 s, *P* = 0.005) and QAC-based (*P* < 0.001) products and the water control (*P* < 0.001; Fig. 3c and d). Relative to the water control, no statistically significant differences were observed for the AAS-based product (30 s, *P* = 0.652; 60 s, *P* = 0.822) or for the QAC-based product (30 s, *P* = 0.131; 60 s, *P* = 0.180). As was the case for the other viruses, extending the contact time to 60 s produced no significant added benefit in efficacy against TuV compared to 30 s (*P* = 0.797; Fig. 3c and d).

**FIG 4:**
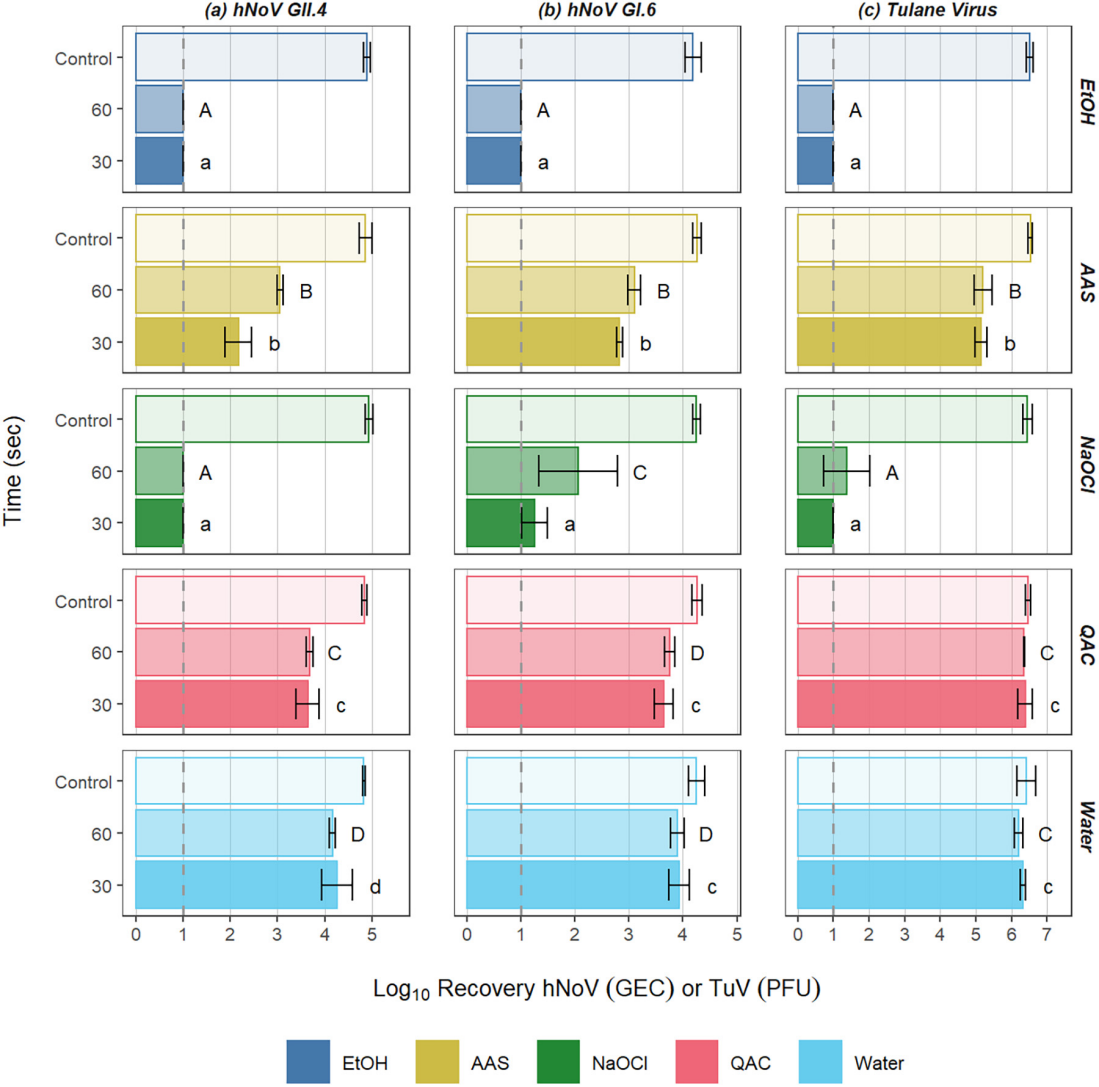
Log_10_ recoveries of GII.4 hNoV GEC (a), GI.6 hNoV GEC (b), and TuV PFU (c) from paper towels following treatment of virus-inoculated Formica surfaces with various surface sanitizers applied with a paper towel wiping step. Controls represent the viral titer of dried inocula on untreated coupons. The dotted line represents the limit of detection of the assay. Different letters within a column of panels represent significant statistical differences (*P* < 0.05) in the log_10_ recovery of virus when comparing sanitizing products, with lowercase letters being used for 30 s and uppercase letters being used for 60 s. EtOH, ethanol; AAS, acid + anionic surfactant; NaOCl, sodium hypochlorite; QAC, quaternary ammonium compound.

When the paper towels were processed for enumeration of residual virus 5 min after wiping, there was increased differentiation between product efficacy. In general, the results were distributed in roughly three tiers, i.e., the lowest recoveries being observed for towels used in application of the EtOH-based and NaOCl-based products, with considerably higher recoveries for AAS-based products, and an additional further increase in recovery for the QAC-based product and water (Fig. 4). For all viruses, no evidence of residual virus could be detected on the used paper towels with the EtOH-based product treatments, suggesting complete inactivation of hNoVs by this product. For the NaOCl-based product, no detectable virus was present on spent paper towels used in wiping studies for GII.4 hNoVs, and relatively low concentrations of virus were recovered from paper towels for GI.6 and TuV (Fig. 4). For the AAS-based product, the concentrations of virus recovered from the paper towels were approximately 2.3, 1.3, and 1.4 log_10_ lower than that of the untreated control coupon for GII.4, GI.6, and TuV, respectively. For the QAC-based product and water, the concentration of virus recovered from the paper towels was similar to that of the initial dried inoculum, suggesting low (if any) virus inactivation by the product.

## DISCUSSION

In this study, controlled surface assays were done to assess the relative anti-hNoV efficacy of four commercially available surface sanitizer chemistries and a water control as applied to a commonly used restaurant tabletop material. The results clearly showed a significant difference in anti-hNoV efficacy of the products tested, with some providing significant anti-hNoV activity while others provided nearly no log_10_ reduction. Controlled experiments were also done to assess the added benefit of incorporating a paper towel wiping step, which is a common practice. The differences in results between the traditional surface assays done using ASTM methods and our newly developed wiping assays suggest that physical removal through wiping is a major contributor to efficacy when included as part of the disinfection process. Further, the high concentrations of virus remaining on the paper towel 5 min after wiping with some of the products raises concerns for transfer and cross-contamination potential by the used paper towel when using formulations that do not demonstrate significant anti-hNoV activity.

For all treatments included in this study, regardless of virus used as inoculum, contact time, or inclusion/exclusion of a wiping step, the EtOH-based product produced a significantly greater reduction in virus concentration on Formica coupons (>3.0 log_10_ hNoV GEC reduction at 30 s without wiping, and >4.0 log_10_ reduction with wiping) compared to the other chemistries (*P* < 0.05; Fig. 1 to 3). While this is only the second set of findings for this particular product (14), the results for the other chemistries are consistent with previous reports (11, 12, 14). For example, NaOCl solutions containing 200 ppm free chlorine have previously been shown to be minimally effective against hNoVs after even a 5-min contact time (12). Poor results for the QAC-based product at 400 ppm are likewise unsurprising due to lack of an antiviral mechanism of action against nonenveloped viruses, and previous studies showing a lack of QAC efficacy in the inactivation of hNoVs and their surrogates (11, 17, 26). The AAS-based product demonstrated incomplete anti-hNoV efficacy likely due to the documented resistance of nonenveloped enteric viruses to acidic pH (12). We chose to evaluate all products at 30- and 60-s contact times. Specifically, the AAS- and EtOH-based products have 30-s disinfection claims against hNoVs based on FCV surrogate data (Table 1), while all the products recommend a 60-s contact time when used on FCS (27). Thirty seconds is also a relevant contact time to evaluate, as shorter contact times are more reflective of real-use applications in the retail food sector (28).

**TABLE 1:** Surface sanitizers evaluated in this study

Product description	Product name	Manufacturer and location	EPA registration no.	EPA-registered norovirus claim^*a*^	Active ingredient(s)	Active ingredient concn of use solution	pH of use solution
Sodium hypochlorite-based sanitizer (NaOCl)	Clorox disinfecting bleach	Clorox Company, Oakland CA	5813-120	NA	Sodium hypochlorite	200 ppm available chlorine	10.5
Quaternary ammonium compound-based sanitizer (QAC)	Oasis 146 multi-quat sanitizer	Ecolab Inc., St. Paul, MN	1677-198	NA	Alkyl (C14, 50%; C12, 40%; C16, 10%) dimethyl benzyl ammonium chloride, octyl decyl dimethyl ammonium chloride, didecyl dimethyl ammonium chloride, dioctyl dimethyl ammonium chloride	400 ppm total quaternary ammonium compounds	7.7
Acid and anionic surfactant-based sanitizer (AAS)	Sink and surface cleaner sanitizer	Ecolab Inc., St. Paul, MN	1677-260	30 s	Dodecylbenzene sulfonic acid and lactic acid	1,875 ppm lactic acid and 700 ppm dodecylbenzene sulfonic acid	2.7
Ethanol-based sanitizer (EtOH)	PURELL foodservice surface sanitizer	GOJO Industries, Akron, OH	84368-1-84150	30 s	Ethanol	29.4% (v/v)	12.8

a Products tested that have Environmental Protection Agency (EPA)-registered claims as food contact surfaces (FCS) sanitizers with efficacy against human noroviruses (hNoVs) based on performance data against feline calicivirus (FCV) (22). NA indicates that products do not carry an EPA-registered norovirus claim.

In this study, the EtOH-based product produced the highest degree of viral inactivation. Many studies have concluded that, regardless of concentration, ethanol is only marginally effective against hNoVs (11, 12, 29), TuV (30, 31), and the commonly used surrogate FCV (12). Nonetheless, in this study, the EtOH-based product showed a high degree of efficacy, which is probably driven by the total product formulation rather than the single active ingredient of ethanol. In particular, the viral capsid protein exposure to the highly basic pH of this product (Table 1) likely contributes significantly to its efficacy by altering the charge state of amino acid side chains, leading to protein unfolding as a result of lost hydrogen bond and salt-bridge interactions. This is supported by previous research in which deformation of hNoV-like particles occurred when exposed to alkaline conditions (32, 33). Given previously observed synergistic antiviral effects of ethanol and high pH for hNoVs (14, 34), the combination of ethanol and high pH is likely the primary driver of this product’s efficacy, resulting in further disruptions to hydrogen bonding in addition to stabilizing hydrophobic residues exposed during protein denaturation. (35). Indeed, the use of pH manipulation to synergistically enhance antiviral efficacy of products has become more common, for example, alkaline QAC solutions (36), acidic hydrogen peroxide product formulations (37), and the combination of citric acid and alcohol for hand antiseptics (34).

Some of the early data used to justify the use of FCV as an appropriate surrogate for hNoVs were based on testing of active ingredients rather than product formulations (38). U.S. EPA approval for surface sanitizer anti-hNoV label claims relies on demonstration of product efficacy against FCV (22), despite the fact that it is a respiratory virus with higher susceptibility to pH extremes and demonstrated susceptibility to chlorine compared to hNoVs (12). This calls into question the reliability of anti-hNoV label claims based on FCV data. Supporting this point is the fact that we observed minimal efficacy with the AAS-based product against hNoVs, despite this product having a registered EPA claim against hNoVs. Given the severe limitations of FCV as a surrogate for hNoVs, it is likely that there are additional EPA-registered products making claims against hNoVs that actually have minimal efficacy, which likely contribute to a false sense of security and elevated public health risk.

Although not used to support U.S. EPA label claims, another widely used surrogate, MNV, has been shown to be more highly susceptible to alcohols than hNoVs (11, 12). The comparative findings reported here, which demonstrate that TuV behavior is similar to that of two hNoV strains for all products tested, support previous observations that TuV may be a more relevant surrogate for approximating anti-hNoV efficacy of antimicrobial surface sanitizers and disinfectants (12). This is particularly important in the absence of hNoV infectivity data. Combining TuV infectivity data with RNase-RT-qPCR data, as was done in this study, should provide added assurance of product efficacy against human strains of norovirus. In the absence of a hNoV infectivity model suitable for routine use for product screening, adoption of better surrogate viruses (for example, TuV as discussed below) should be considered by regulatory authorities to ensure that products registered as having anti-hNoV properties are truly effective against human strains of the virus.

For all products tested, the addition of a wiping step significantly improved surface sanitizer efficacy. Although the EtOH-based product showed a high degree of anti-hNoV activity even without wiping, the addition of a wiping step for the other chemistries increased anti-hNoV efficacy from ≤0.5 log_10_ reduction to 1.6 to 3.8 log_10_ reduction, dependent on the product and the virus. The significant improvement in results for products that previously showed minimal anti-hNoV activity by standard surface assay supports the importance of wiping in the sanitization process as a whole. While previously published studies on the efficacy of removal by wiping with a substrate (e.g., cloth, paper towel, etc.) are scarce, our findings are in line with those of Gibson et al. (24), who evaluated removal of hNoVs and several surrogate viruses from similar tabletop materials. In that study, various cleaning cloths (cotton, microfiber, terry towel, among others) were shown to efficiently remove these viruses from stainless steel surfaces, with greater than 2 log_10_ reductions of virus observed following wiping using the cloths dampened with water only (24).

Interesting results were observed when evaluating how much virus remained on the used paper towels 5 min after wiping. Based on the nearly complete inactivation of virus by the EtOH-based product in the ASTM surface assays, one would not expect to recover significant virus from towels following wiping with this product. Sanitization in the case of this product seems to be driven predominantly by virus inactivation. After wiping with water or the QAC-based product, high concentrations of virus could be recovered from used paper towels, with nearly the full 6 log_10_ PFU starting inoculum recovered for TuV and approximately 3 to 4 log_10_ hNoV GEC recovered. In this case, the efficacy of sanitization appears to be almost completely driven by physical removal rather than virus inactivation. These data are roughly in line with those previously published using other wiping methods (24, 39). Approximately 1 log_10_ less virus concentration was recovered from the used paper towels after treatment with the AAS-based product compared to the QAC-based product and water control, suggesting a combination of virus removal and inactivation. The same can be said for the NaOCl-based product, but in this case, very little virus was recovered from the towels, despite the fact that the product itself did not produce notable reduction in virus concentration in the standard ASTM surface assays performed without wiping. In this case, it is possible that spray application followed by mechanical action during the wiping process itself may assist in resuspension of the viral matrix. This then facilitates distribution of virus particles across the paper towel surface, making them more accessible to chemistries that have shown some efficacy against hNoVs (e.g., sodium hypochlorite) while not influencing the efficacy of chemistries that have shown minimal efficacy against hNoVs (e.g., AAS and QAC). This mechanism could potentially be enhanced by other components in the formulation, including surfactants, which contribute to cleaning ability and can influence spread behavior of the product to aid in more complete coverage of the spray area (40). We visually observed increased friction between the paper towel and the test surface with the water and NaOCl-based product, presumably due to their lack of surfactants. When standardized for the same wiping pressure, increased friction may also be driving the efficiency of the removal process for products without surfactants; however, this observation may not translate into real-life wiping conditions in which human behavior may result in altered pressure applied in response to perceived friction.

The wiping method developed in this study was designed to simulate realistic use conditions while controlling as many variables as possible. This was done by combining the attributes of the initial EPA method of spray application (41) for a defined contact time, upon which we added an additional wiping step prior to neutralization. The method utilizes an automated wiping action representative of typical behavior (left-to-right, back-and-forth motion), which gives increased consistency in wiping, while retaining the use of small coupons and small volumes of virus inoculum, as used with the Wiperator, to allow for easy elution of virus without swabbing. A larger surface area is used to accommodate spray application and horizontal movement for the wiping action, similar to the 4-field test, to better simulate full scale wiping. Variables such as wiping pressure applied, wetness of the paper towel, physical properties of the paper towel, and number of wiping passes were all considered while developing the method (data not shown). The choice of mass to provide the downward wiping force was carefully considered in context of the wiping surface area to achieve a wiping pressure (force/surface area) on par with the unitary mass used in the 4-field test. Prewetting of the paper towel by spraying and wiping (before treating the virus-inoculated surface via spraying) was utilized to more closely mimic real-life wiping conditions. Formica coupons were used instead of glass or stainless steel, as this is a commonly used tabletop surface in restaurants, a major setting for hNoV outbreaks. Preliminary testing (data not shown) demonstrated minimal differences between two, four, and six wiping passes, so six passes were used to more closely align with the EPA method (42) and give the sanitizers tested higher potential efficacy.

The results of this study highlight the great variability in anti-hNoV activity of representative surface sanitizers, in some cases, despite having label claims specifying this activity. Individuals evaluating antimicrobial surface products for hNoV efficacy should seek products with additional predictive testing data (e.g., hNoV data, additional surrogate viruses such as TuV) that support their claims. This study also highlights the importance of wiping in the overall process of sanitization. This study supports the importance of proper disposal of paper towels used during cleaning up diarrheal or vomiting incidents suspected to be caused by hNoVs. Lack of proper disposal of these spent paper towels could result in cross-contamination. Although evaluating cross-contamination potential was outside the scope of this work, this could be done using the same protocol developed in this study, as could evaluation of product performance on other surface materials such as glass or stainless steel. In the meantime, for products that do not show efficacy or otherwise lack a mechanism of action against nonenveloped enteric viruses, care should be taken to avoid spreading infectious virus to other surfaces during wiping, a phenomenon that has been demonstrated using hNoV surrogates (24). There is a clear need to develop surface sanitizers and disinfectant formulations with demonstrated efficacy against hNoVs, which may necessitate the use of alternative surrogates such as TuV. Findings from this study are valuable to food safety personnel in restaurant settings, as they help these individuals make informed decisions on product selection and application methods in order to reduce the risk of hNoV contamination and transmission in their facilities.

## MATERIALS AND METHODS

### Virus stocks.

Human stool specimens obtained from outbreaks of hNoVs GII.4 Sydney and GI.6 (provided courtesy of Shermalyn Greene, North Carolina State Laboratory of Public Health, Raleigh, NC) were used as inoculum. They were prepared as 20% dilutions in phosphate-buffered saline (PBS) followed by clarification to remove solids via centrifugation (10,000 × *g* for 10 min at 4°C). The stock titer for GII.4 Sydney was 8.7 log_10_ genome equivalent copies (GECs)/mL and 7.8 log_10_ GECs/mL for GI.6.

Tulane virus (TuV) stock was prepared by passaging in LLC-MK2 cells (both provided courtesy of Xi Jiang, Cincinnati Children’s Hospital Medical Center, Cincinnati, OH) using cell culture media and incubation conditions described previously (23). Briefly, LLC-MK2 cells were grown to 90% confluence in T175 flasks (Eppendorf, Hamburg, Germany) and then infected with TuV at a multiplicity of infection of 0.8 for 11 h. The cells were harvested with a cell scrapper and centrifuged to discard cell culture media, and the pellet was resuspended in 350 μL PBS per T175 flask. The cells were subjected to three freeze-thaw cycles, and virus was purified by solvent extraction with Vertrel XF (Chemours, Wilmington, DE) and further purified using two sequential passages through a Capto-Core 700 resin (Cytiva, Marlborough, MA) utilizing a slurry approach as previously described (43). This yielded a semipurified virus stock to which fetal bovine serum (FBS; Thermo Fisher, Waltham, MA) was added to achieve a soil load equivalent to approximately 5% FBS (44). The final TuV virus stock titer was 8.2 log_10_ PFU/mL.

### Preparation of surface test materials.

Coupons were prepared from a sheet of Formica-branded laminate composite tabletop material (Formica Group, Cincinnati, OH) cut into small rectangles (12.8 ± 0.3 mm × 50 ± 0.5 mm). Autoclave sterilization was not possible due to irreversible damage to coupons caused by the extreme heat and pressure, so they were disinfected by wiping with 90% ethanol, followed by air drying for 10 min prior to use. All coupons were used once and discarded after autoclaving. The acrylic inserts used in the wiping protocol were disinfected for 10 min in a 5,000 ppm sodium hypochlorite bath, rinsed in distilled water, and washed in a dishwasher using Contrad NF (Decon Laboratories, King of Prussia, PA) liquid detergent to remove any lingering organic matter or disinfectant chemicals. The inserts were wiped with 90% ethanol prior to use to eliminate any residual surfactants remaining from washing.

### Products screened.

Four commercially available surface sanitizers were evaluated in this study. Product characteristics, including manufacturer information, EPA registration numbers, active ingredients, and use concentrations are provided in Table 1. Surface sanitizers that required dilution to a working concentration were freshly prepared on the same day as the experimental replicate by dilution in distilled water according to manufacturer’s instructions. The sodium hypochlorite-based surface sanitizer (NaOCl) was diluted to 200 ppm free chlorine based on measured total chlorine content via iodometric method with a digital titrator (Hach Co, Loveland, CO). NaOCl- and QAC-based products were tested at the highest concentration of active ingredients allowed for use on food contact surfaces per federal guidelines, at 200 and 400 ppm, respectively (15). These commonly used classes of FCS sanitizers served as a point of reference for the acid and anionic surfactant (AAS)-based product and the ethanol (EtOH)-based product, both of which provide label claims against hNoVs. The EtOH-based product was used as supplied by the manufacturer in its ready-to-use form, while the AAS-based product was used at the highest manufacturer-recommended concentration for inactivating hNoVs on FCS (i.e., 1,875 ppm lactic acid and 700 ppm dodecylbenzenesulfonic acid). Additional product characteristics, including safety considerations, are listed in Table S1. In addition to the four test products, sterile distilled water alone was included as an additional control.

### Neutralization of sanitizers.

Choice of neutralizer was dependent upon virus tested. For testing against hNoVs, the products were neutralized using PBS supplemented with 10% beef extract (Thermo Fisher), 0.04% Tween 80 (Sigma, St. Louis, MO), and 0.2% sodium thiosulfate (Sigma). The neutralizer for TuV testing was M199 media (Corning, Corning, NY), supplemented with 10% FBS and 0.2% sodium thiosulfate. M199 was further modified by addition of HCl when evaluating the EtOH-based product to achieve a pH of ~7 to 8 following product neutralization.

### Virucidal surface assays.

Virucidal surface assays were performed in accordance with the ASTM E1053-20 protocol (44), with minor modifications for inoculum volume, coupon type and size, and elution method. Briefly, the coupons were inoculated with 20 μL of virus inoculum (representing approximately 7.0 log_10_ GEC for GII.4 Sydney, 6.1 log_10_ GEC for GI.6, and 6.5 log_10_ PFU for TuV), spread to approximately 0.5 cm^2^, and allowed to fully dry in a BSL-2 hood before sanitizer application (90 to 120 min). Subsequently, the coupons were treated with 180 μL of the surface sanitizer and held for contact times of 30 and 60 s. The coupons were then aseptically transferred to a 15-mL conical tube containing 1.8 mL of neutralizing buffer. Product neutralization and virus elution were performed by vortexing for 60 s. As per ASTM, neutralization controls were done by treating a virus-inoculated coupon with a 1:10 dilution of the respective sanitizer in neutralizing buffer for 60 s. Negative controls consisted of uninoculated coupons treated with neutralizer for 60 s. Eluates from the hNoV experiments, including all controls, were subjected to RNase pretreatment to destroy unencapsidated or otherwise unprotected RNA. For the RNase pretreatment, 2 μL RNase One (Promega, Madison, WI) along with 22 μL of reaction buffer were added to 200 μL of the eluate and incubated at 37°C for 15 min. The samples were then placed on ice for 5 min to halt the RNase enzyme digestion. RNase-treated eluates were stored at –80°C until RNA extraction and quantification. The eluates from the TuV trials were not stored on ice but instead were immediately serially diluted in PBS. These serial dilutions were then inoculated onto prepared LLC-MK2 plates for plaque assay-based TuV quantification.

### Wiping studies.

Wiping was performed using a modified Gardner-scrub abrasion testing machine, configured with the ISO arm adapter and pad holder (Gardco, Pompano Beach, FL). The arm adapter was modified to change the orientation of the wiping head and to ensure the height was sufficient, so it facilitated only horizontal movement of the wiping assembly head, with no additional downward force applied. A 450 × 170 × 6-mm acrylic insert was used as the wiping surface, with a 51 × 13.5 × 1-mm slot centered on the left side to hold a virus-inoculated Formica coupon (Fig. S1). Tork W24 paper towels (Essity, Philadelphia, PA) were used as the wiping substrate and were prepared by folding three times and trimming to fit the pad holder in a virus-free area with sterile scissors (Fig. S2). The wiping head was assembled by wrapping a paper towel around the pad holder, with a Scotch-Brite pad (number 7448, 3M, St. Paul, MN) sandwiched in the middle, using paper clips to secure the paper towel (Fig. S3A and B). The wiping head was placed on the acrylic insert with a 500-g mass resting on top, and the rods of the pad holder were positioned in the slots of the scrubber arm adapter (Fig. S4A and B). The wiping head, including pad holder, pad, and paper towel, had a combined mass of 133 g, resulting in a total mass of 633 g used for the downward wiping force, applied over an effective contact surface area of 90 × 39 mm between the paper towel and the wiping surface.

To control for variability in sprayer type, spray pattern, and spray volume, identical commonly used polyethylene spray bottles (Homestead Choice LLC, Dover, DE) were acquired from an online marketplace and used for spray application of all products, regardless of how the product was supplied by the manufacturer. The coupons were prepared and inoculated as described for the standard surface assay above. Likewise, the wiping apparatus was setup for testing as described above. Immediately prior to testing, the acrylic insert surface was first sprayed with 4 mL of test sanitizer, and the surface was wiped twice to collect the fluid and premoisten the paper towel. The virus-inoculated coupon was placed in the recessed slot of the acrylic insert, and 1 mL of sanitizer was sprayed over the coupon, at a 45° angle approximately 20 cm away from the surface. Spray application resulted in approximately 80 μL of sanitizer applied directly to the coupon, and regardless of product or virus tested, the spray application resulted in the entire viral inoculum region of the coupon being covered with sanitizer. After the prescribed contact time, the machine performed a total of six wiping passes (three back-and-forth wipes) of the paper towel over the coupon, followed by immediate transfer of the coupon to a 15-mL conical tube with 2 mL of neutralization buffer. The coupon was vortexed for 60 s to neutralize the product and elute the remaining virus. The paper towel was sampled 4.5 to 5 min after the start of the wiping action. To do this, the cross-sectional area of the paper towel that contacted the coupon was cut using sterile scissors, followed by vortexing for 60 s in a 50-mL conical tube containing 20 mL of the appropriate neutralization buffer. The paper towel eluates and controls were processed for virus detection and quantification in a manner identical to that used for the surface eluates.

All of the instruments used for manipulation of coupons and paper towels were flame-sterilized between each coupon, and the acrylic insert was swapped for a clean, sterilized insert for each experimental replication. During wiping experiments, double gloving was utilized for assembly and preparation of the wiping assembly head to minimize risk of cross-contamination to paper towels and laboratory surfaces. The entire machine and work area were sterilized with a 5,000 ppm solution of sodium hypochlorite at the end of experiments each day.

### RNA extraction and RT-qPCR for hNoVs.

Eluates from the hNoV studies were extracted for RNA isolation using the NucliSENS EasyMag system (bioMérieux, Durham, NC) with a final elution volume of 25 μL in proprietary buffer. Viral RNA detection was done by RT-qPCR targeting the conserved ORF1-ORF2 junction using primers QNIFS and COG2R and probe QNIF2d for hNoV GII; and primers COG1F and COG1R and probes RING1(a) and RING1(b) for hNoV GI (45, 46). RNA standards representing the GII.4 and GI.6 strains used in this study were prepared as a series of 1:10 dilutions and used to construct standard curves correlating cycle threshold (*C_T_*) values to log_10_ GEC input values. For quantification, the resulting cycle threshold (*C_T_*) values were extrapolated to log_10_ GEC using the linear regression derived from the standard curve. Log_10_ reduction of hNoV GEC was determined by subtraction from the value obtained for the respective neutralized control coupon.

### Infectivity assay for TuV.

Tulane virus infectivity was determined by plaque assay using LLC-MK2 cells (23). Due to the observed cytotoxicity for the EtOH-based product, all controls and treatments for this product were passed through a Pierce detergent removal column (Thermo Fisher) immediately after neutralization. Log_10_ reduction in TuV infectivity was calculated as the difference between the respective neutralization control and treatment log_10_ PFU. The LOD was calculated as the difference between the neutralization control coupon and the plate corresponding to the least diluted sample for which cytotoxicity was absent.

### Statistical analysis.

Three experimental replications were performed for each product, virus, and time point. The results are expressed as means ± standard deviation of log_10_ GEC reduction for hNoVs or log_10_ PFU reduction for TuV. The data were analyzed using R version 4.1.1 (R Core Team, 2021, Vienna, Austria) with statistical comparison performed via factorial analysis of variance (ANOVA) followed by Tukey honestly significant difference (HSD) *post hoc* test. Statistical significance was established at a level of *P < *0.05.

## References

[1] Pires SM, Fischer-Walker CL, Lanata CF, Devleesschauwer B, Hall AJ, Kirk MD, Duarte ASR, Black RE, Angulo FJ. 2015. Aetiology-specific estimates of the global and regional incidence and mortality of diarrhoeal diseases commonly transmitted through food. PLoS One 10:e0142927. 10.1371/journal.pone.0142927. 26632843 PMC4668836

[2] Bartsch SM, Lopman BA, Ozawa S, Hall AJ, Lee BY. 2016. Global economic burden of norovirus gastroenteritis. PLoS One 11:e0151219. 10.1371/journal.pone.0151219. 27115736 PMC4846012

[3] Scallan E, Hoekstra RM, Angulo FJ, Tauxe RV, Widdowson M-A, Roy SL, Jones JL, Griffin PM. 2011. Foodborne illness acquired in the United States—major pathogens. Emerg Infect Dis 17:7–15. 10.3201/eid1701.P11101. 21192848 PMC3375761

[4] Simmons K, Gambhir M, Leon J, Lopman B. 2013. Duration of immunity to norovirus gastroenteritis. Emerg Infect Dis 19:1260–1267. 10.3201/eid1908.130472. 23876612 PMC3739512

[5] Esposito S, Principi N. 2020. Norovirus vaccine: priorities for future research and development. Front Immunol 11:1383. 10.3389/fimmu.2020.01383. 32733458 PMC7358258

[6] Hall AJ, Lopman BA, Payne DC, Patel MM, Gastañaduy PA, Vinjé J, Parashar UD. 2013. Norovirus disease in the United States. Emerg Infect Dis 19:1198–1205. 10.3201/eid1908.130465. 23876403 PMC3739528

[7] Teunis PFM, Moe CL, Liu P, Miller SE, Lindesmith L, Baric RS, Pendu JL, Calderon RL. 2008. Norwalk virus: how infectious is it? J Med Virol 80:1468–1476. 10.1002/jmv.21237. 18551613

[8] Atmar RL, Opekun AR, Gilger MA, Estes MK, Crawford SE, Neill FH, Ramani S, Hill H, Ferreira J, Graham DY. 2014. Determination of the 50% human infectious dose for Norwalk virus. J Infect Dis 209:1016–1022. 10.1093/infdis/jit620. 24253285 PMC3952671

[9] Atmar RL, Opekun AR, Gilger MA, Estes MK, Crawford SE, Neill FH, Graham DY. 2008. Norwalk virus shedding after experimental human infection. Emerg Infect Dis 14:1553–1557. 10.3201/eid1410.080117. 18826818 PMC2609865

[10] Lopman B, Gastañaduy P, Park GW, Hall AJ, Parashar UD, Vinjé J. 2012. Environmental transmission of norovirus gastroenteritis. Curr Opin Virol 2:96–102. 10.1016/j.coviro.2011.11.005. 22440972

[11] Tung G, Macinga D, Arbogast J, Jaykus L-A. 2013. Efficacy of commonly used disinfectants for inactivation of human noroviruses and their surrogates. J Food Prot 76:1210–1217. 10.4315/0362-028X.JFP-12-532. 23834796

[12] Cromeans T, Park GW, Costantini V, Lee D, Wang Q, Farkas T, Lee A, Vinjé J. 2014. Comprehensive comparison of cultivable norovirus surrogates in response to different inactivation and disinfection treatments. Appl Environ Microbiol 80:5743–5751. 10.1128/AEM.01532-14. 25015883 PMC4178592

[13] U.S. Centers for Disease Control and Prevention. 2021. Norovirus: preventing norovirus. https://www.cdc.gov/norovirus/about/prevention.html.

[14] Escudero‐Abarca BI, Goulter RM, Bradshaw J, Faircloth J, Leslie RA, Manuel CS, Arbogast JW, Jaykus L. 2022. Efficacy of an alcohol‐based surface disinfectant formulation against human norovirus. J Appl Microbiol 132:3590–3600. 10.1111/jam.15479. 10.1111/jam.15479. 35137492 PMC9306916

[15] U.S. Food and Drug Administration. 2022. CFR — code of federal regulations title 21. https://www.accessdata.fda.gov/scripts/cdrh/cfdocs/cfcfr/CFRSearch.cfm?FR=178.1010.

[16] Bolton SL, Kotwal G, Harrison MA, Law SE, Harrison JA, Cannon JL. 2013. Sanitizer efficacy against murine norovirus, a surrogate for human norovirus, on stainless steel surfaces when using three application methods. Appl Environ Microbiol 79:1368–1377. 10.1128/AEM.02843-12. 23263949 PMC3568589

[17] Feliciano L, Li J, Lee J, Pascall MA. 2012. Efficacies of sodium hypochlorite and quaternary ammonium sanitizers for reduction of norovirus and selected bacteria during ware-washing operations. PLoS One 7:e50273. 10.1371/journal.pone.0050273. 23227163 PMC3515596

[18] Hoelzer K, Fanaselle W, Pouillot R, Doren JMV, Dennis S. 2013. Virus inactivation on hard surfaces or in suspension by chemical disinfectants: systematic review and meta-analysis of norovirus surrogates. J Food Prot 76:1006–1016. 10.4315/0362-028X.JFP-12-438. 23726196

[19] Shimizu-Onda Y, Akasaka T, Yagyu F, Komine-Aizawa S, Tohya Y, Hayakawa S, Ushijima H. 2013. The virucidal effect against murine norovirus and feline calicivirus as surrogates for human norovirus by ethanol-based sanitizers. J Infect Chemother 19:779–781. 10.1007/s10156-012-0516-2. 23135829

[20] Park GW, Sobsey MD. 2011. Simultaneous comparison of murine norovirus, feline calicivirus, coliphage MS2, and GII.4 norovirus to evaluate the efficacy of sodium hypochlorite against human norovirus on a fecally soiled stainless steel surface. Foodborne Pathog Dis 8:1005–1010. 10.1089/fpd.2010.0782. 21457050

[21] Knight A, Li D, Uyttendaele M, Jaykus L-A. 2013. A critical review of methods for detecting human noroviruses and predicting their infectivity. Crit Rev Microbiol 39:295–309. 10.3109/1040841X.2012.709820. 22900992

[22] U.S. Environmental Protection Agency. 2018. Product performance test guideline, OCSPP 810.2000, general considerations for testing public health antimicrobial pesticides, guidance for efficacy testing [EPA 712-C-17–002]. https://www.regulations.gov/document/EPA-HQ-OPPT-2009-0150-0034.

[23] Farkas T, Sestak K, Wei C, Jiang X. 2008. Characterization of a rhesus monkey calicivirus representing a new genus of Caliciviridae. J Virol 82:5408–5416. 10.1128/JVI.00070-08. 18385231 PMC2395209

[24] Gibson KE, Crandall PG, Ricke SC. 2012. Removal and transfer of viruses on food contact surfaces by cleaning cloths. Appl Environ Microbiol 78:3037–3044. 10.1128/AEM.00027-12. 22327573 PMC3346440

[25] Jacobshagen A, Gemein S, Exner M, Gebel J. 2020. Test methods for surface disinfection: comparison of the Wiperator ASTM standard E2967-15 and the 4-field test EN 16615. Gms Hyg Infect Control 15:Doc04. 32547904 10.3205/dgkh000339 PMC7273320

[26] Lee J-W, Kang L-H, Kim M-K, Kim J-S, Kim ML, Lee S-G, Choi I-H, Park C-J, Paik S-Y. 2021. Determining the efficacy of 27 commercially available disinfectants against human noroviruses. J Infect Public Health 14:244–248. 10.1016/j.jiph.2020.12.004. 33493921

[27] U.S. Environmental Protection Agency. 1979. Label requirements for pesticides used for sanitation of food contact surfaces. https://www.epa.gov/sites/default/files/2020-04/documents/label_requirements_for_pesticides_used_for_sanitation_of_food_contact_surfaces.pdf.

[28] Howells AD, Roberts KR, Shanklin CW, Pilling VK, Brannon LA, Barrett BB. 2008. Restaurant employees’ perceptions of barriers to three food safety practices. J Am Diet Assoc 108:1345–1349. 10.1016/j.jada.2008.05.010. 18656574

[29] Costantini V, Morantz EK, Browne H, Ettayebi K, Zeng X-L, Atmar RL, Estes MK, Vinjé J. 2018. Human norovirus replication in human intestinal enteroids as model to evaluate virus inactivation. Emerg Infect Dis 24:1453–1464. 10.3201/eid2408.180126. 30014841 PMC6056096

[30] Tian P, Yang D, Quigley C, Chou M, Jiang X. 2013. Inactivation of the Tulane virus, a novel surrogate for the human norovirus. J Food Prot 76:712–718. 10.4315/0362-028X.JFP-12-361. 23575140 PMC4073237

[31] Arthur SE, Gibson KE. 2015. Physicochemical stability profile of Tulane virus: a human norovirus surrogate. J Appl Microbiol 119:868–875. 10.1111/jam.12878. 26104882

[32] Cuellar JL, Meinhoevel F, Hoehne M, Donath E. 2010. Size and mechanical stability of norovirus capsids depend on pH: a nanoindentation study. J Gen Virol 91:2449–2456. 10.1099/vir.0.021212-0. 20592107

[33] Pogan R, Schneider C, Reimer R, Hansman G, Uetrecht C. 2018. Norovirus-like VP1 particles exhibit isolate dependent stability profiles. J Phys Condens Matter 30:e064006. 10.1088/1361-648X/aaa43b. PMC7104913 29282349

[34] Sato S, Matsumoto N, Hisaie K, Uematsu S. 2020. Alcohol abrogates human norovirus infectivity in a pH-dependent manner. Sci Rep 10:15878. 10.1038/s41598-020-72609-z. 32985508 PMC7522253

[35] Yoshizawa S, Arakawa T, Shiraki K. 2014. Dependence of ethanol effects on protein charges. Int J Biol Macromol 68:169–172. 10.1016/j.ijbiomac.2014.04.041. 24780565

[36] Shirai J, Kanno T, Inoue T, Mitsubayashi S, Seki R. 1997. Effects of quaternary ammonium compounds with 0.1% sodium hydroxide on swine vesicular disease virus. J Vet Med Sci 59:323–328. 10.1292/jvms.59.323. 9192351

[37] Omidbakhsh N, Sattar SA. 2006. Broad-spectrum microbicidal activity, toxicologic assessment, and materials compatibility of a new generation of accelerated hydrogen peroxide-based environmental surface disinfectant. Am J Infect Control 34:251–257. 10.1016/j.ajic.2005.06.002. 16765201 PMC7132737

[38] Doultree JC, Druce JD, Birch CJ, Bowden DS, Marshall JA. 1999. Inactivation of feline calicivirus, a Norwalk virus surrogate. J Hosp Infect 41:51–57. 10.1016/s0195-6701(99)90037-3. 9949965

[39] Becker B, Henningsen L, Paulmann D, Bischoff B, Todt D, Steinmann E, Steinmann J, Brill FHH, Steinmann J. 2019. Evaluation of the virucidal efficacy of disinfectant wipes with a test method simulating practical conditions. Antimicrob Resist Infect Control 8:121. 10.1186/s13756-019-0569-4. 31346462 PMC6636036

[40] Kovalchuk NM, Simmons MJH. 2021. Surfactant-mediated wetting and spreading: recent advances and applications. Curr Opin Colloid Interface Sci 51:101375. 10.1016/j.cocis.2020.07.004.

[41] U.S. Environmental Protection Agency. 2021. Standard operating procedure for germicidal spray products as disinfectants (GSPT): testing of *Staphylococcus aureus*, *Pseudomonas aeruginosa*, and *Salmonella enterica*. https://www.epa.gov/sites/default/files/2021-04/documents/mb-06-10.pdf.

[42] U.S. Environmental Protection Agency. 2022. Pre-saturated or impregnated towelettes confirmatory virucidal effectiveness test. https://www.epa.gov/sites/default/files/2015-09/documents/fcv4_towel_confirm_pcol.pdf.

[43] James KT, Cooney B, Agopsowicz K, Trevors MA, Mohamed A, Stoltz D, Hitt M, Shmulevitz M. 2016. Novel high-throughput approach for purification of infectious virions. Sci Rep 6:36826. 10.1038/srep36826. 27827454 PMC5101806

[44] ASTM International. 2021. ASTM 1053-20: standard practice to assess virucidal activity of chemicals intended for disinfection of inanimate, nonporous environmental surfaces. https://webstore.ansi.org/Standards/ASTM/ASTME105320.

[45] Loisy F, Atmar RL, Guillon P, Cann PL, Pommepuy M, Guyader FSL. 2005. Real-time RT-PCR for norovirus screening in shellfish. J Virol Methods 123:1–7. 10.1016/j.jviromet.2004.08.023. 15582692

[46] Kageyama T, Kojima S, Shinohara M, Uchida K, Fukushi S, Hoshino FB, Takeda N, Katayama K. 2003. Broadly reactive and highly sensitive assay for Norwalk-like viruses based on real-time quantitative reverse transcription-PCR. J Clin Microbiol 41:1548–1557. 10.1128/JCM.41.4.1548-1557.2003. 12682144 PMC153860

