# Supplementary material for: The Efficacy of Commercial Surface Sanitizers against Norovirus on Formica Surfaces with and without Inclusion of a Wiping Step
Source: Appl Environ Microbiol. 2022 Aug 25;88(17):e00807-22. doi: 10.1128/aem.00807-22 (PMC9469706; doi:10.1128/aem.00807-22)
Supplement: Supplemental file 1 — Fig. S1 to S5 and Table S1. Download aem.00807-22-s0001.pdf, PDF file, 7.3 MB [file aem.00807-22-s0001.pdf]

	WT	*Δrnf1*	*Δfix*
	Generation Time (hrs)		
19.4 % O_2 (atm)	3.1 ± 0.0	3.0 ± 0.1	4.0 ± 0.0
10% O_2	4.9 ± 0.1	4.5 ± 0.1	8.7 ± 0.2

Supplementary Table 1. Generation times of WT *Δrnf1 and Δfix* strains grown in nitrogen-fixing conditions in a six-well plate. Plates were shaken at 300 RPM in a double orbital rotation and atmosphere was controlled with added nitrogen. (Replicates n =3)

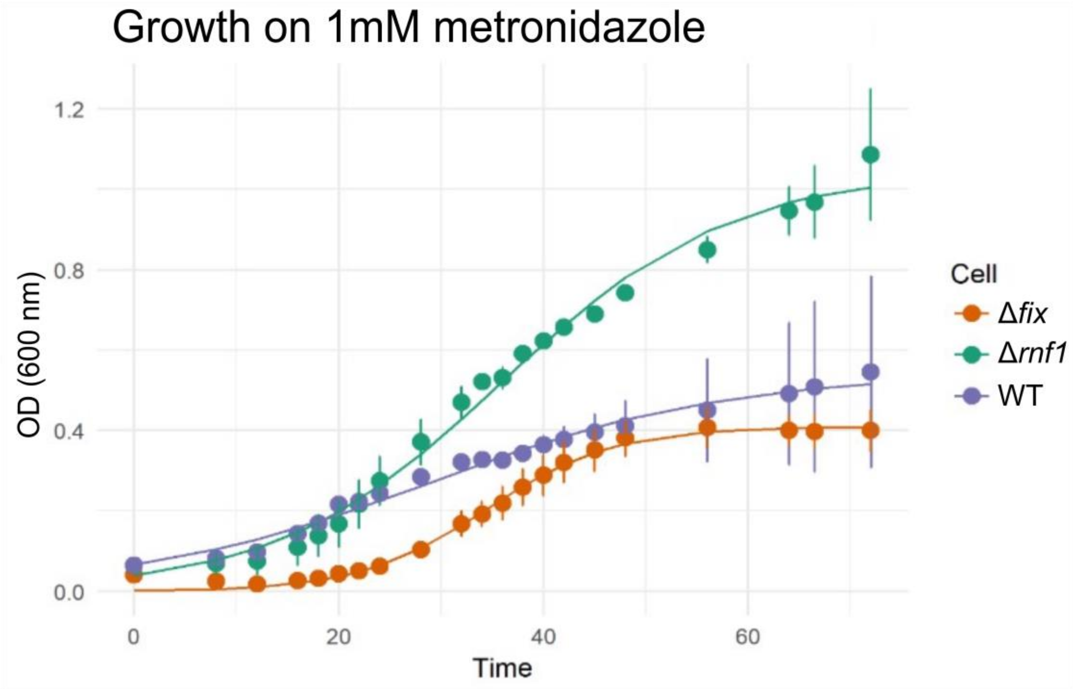

Supplementary Figure 1. Growth rates on 1 mM of metronidazole. WT, Δ*rnf1* and Δ*fix* strains were all grown in a 200 mL of nitrogen free burks media in a 500 mL baffled flask at 200 RPM. Wt and the Δ*fix* strains grow slowly and both do not reach normal terminal ODs. The Δ*rnf1* strain also grows slower than in no metronidazole but is able to reach a higher terminal OD. (Replicates n =3)

Rnf stoichometry is dependent on chemiosmotic potential well as substrate and product mid point potential.

I have used the book Bioenergetics 4 (2013) by David Nicholls and Stuart Ferguson as my main reference for all equations

Rnf has only been characterized biochemically as a Na pump in acetogens, The authors determined a stoichiometry of 2 Na^+ / electrons from reduced ferredoxin (Fd) to NADH. While Rnf has not been characterized in aerobic metabolism where it most likely uses the consumption of protons to facilitate the reduction of Fd from NADH. By determining the stoichiometry we can see how Rnf interacts with the whole electron transport system. (Below)

To determine the effect of the $\frac{NAD^+}{NADH}$ ratio has on the thermodynamics of Rnf we will calculate the mid-point potential of $\frac{NAD^+}{NADH}$ using the Nerst equation:

$$E = E^{o'} + 2.3\frac{RT}{nF}log_{10}\left(\frac{[oxidised]}{[reduced]}\right)$$

Where R is the gas constant, F is the Faraday constant

To utilize the Nerst equation we will use the $\frac{NAD^+}{NADH}$ ratio from Figure 2B:

Strain	$\frac{NAD^+}{NADH}$
Wt	2.7
$\Delta rnf1$	9.8
Δfix	2.35

For $\frac{NAD^+}{NADH}$ redox couple $E^{o'} = -320mv$, but for the nerst equation allowing for the actual cellular redox potential of $\frac{NAD^+}{NADH}$ to be determine by this equation:

$$E = -320 + 2.3\frac{0.008134 \cdot 303}{2 \cdot 96.5}log_{10}(Q)$$

Where Q is the $\frac{NAD^+}{NADH}$ ratio.

We can use python to calculate the above equation and calculate the cellular redox potential for any ratio of $\frac{NAD^+}{NADH}$:

In [1]:

```
#First import the following packages
import math
import numpy as np
import matplotlib.pyplot as plt
```

In [3]:

```
#give physical constants
R = 0.008314 #Gas constant (KJ/ K mol)
T = 303 #Temp at 30C (K)
F = 96.5 #Faraday (KJ / volt gram eq)

#define the nest function
def nerst(E_m7, n, Q):
    #equation in volts so -230 mv is -0.23 v
    return  E_m7 + (2.3*(R*T)/(n*F)) * math.log10(Q)
    #print(E)
```

In [4]:

```
#determine the midpoinit potenital at a 3.0 NAD/NADH ratio
nerst(-0.320, 2,  3.0)
```

Out[4]:

```
-0.3056764080763156
```

In [5]:

```
#determine the midpoinit potenital at a 10 NAD/NADH ratio
nerst(-0.320, 2,  10)
```

Out[5]:

```
-0.28997913678756476
```

So we can see that while the $\frac{NAD^+}{NADH}$ does effect the mid point potential only in the extremes is it going to cause some reactions to be infeasible or something like NADH reducing ferredoxin or flavodoxin which have a mid point potential around -500 mv.

These answers from above now allow us to ask what the ΔG of the Rnf reaction.

First lets lay out the stoichiometry of the reaction:

$$NADH + 2Fd_{ox} + nH_p^+ -> NAD^+ + 2Fd_{red} + nH_c^+$$

Where H_p^+ is periplasm proton and nH_c^+ is the cytoplasm proton and n is the number of protons translocated.

For the redox reaction between NADH and Fd we must calculate ΔE_h:

$$\Delta E_h = E_{h(A)} - E_{h(B)}$$

Where the midpoint potential of Flavodoxin is -483 mv (Segal et al 2016), so ΔE_h is:

$$\Delta E_h = -178mv\left(\frac{NAD^+}{NADH} = 3\right)$$

$$\Delta E_h = -194mv\left(\frac{NAD^+}{NADH} = 10\right)$$

Then to calculate ΔG we use:

$$\Delta G = -nF\Delta E_h$$

$$\Delta G = -2 * (96.5) * (-0.178)$$

$$\Delta G = -2 * (96.5) * (-0.194)$$

Giving:

$$\Delta G = +34.3\frac{kJ}{mol}\left(\frac{NAD^+}{NADH} = 3\right)$$

$$\Delta G = +37.44\frac{kJ}{mol}\left(\frac{NAD^+}{NADH} = 4.35\right)$$

The ΔG is positive as Fd is at a lower mid point potential than NADH hence the need for proton motive force to drive this reaction forward.

The proton motive force $\Delta p\ /pmf$ was measured once in *A. vinelandii* in Laane et al (1980) and present as the electric potential $\Delta\psi$ at $106\ mv$ and the change in pH ΔpH at $0.45\ pH$. We can calculate the proton motive force in $\Delta\mu_{H^+}$ for $\frac{KJ}{mol}$ or Δp for mv through the following equations:

$$\Delta\mu_{H^+} = -F\Delta\Psi + 2.3RT\Delta pH$$

$$\Delta\mu_{H^+}\left(\frac{kJ}{mol}\right) = -96.5\left(\frac{kJ}{mol}\right) + 2.3 * 8.315 * 10^{-3}\left(\frac{kJ}{mol * K}\right) * 298(K) * -0.45(\Delta pH)$$

$$\Delta\mu_{H^+} = -12.78\left(\frac{kJ}{mol}\right)$$

$$\Delta p = \frac{-\Delta\mu_{H^+}}{F}$$

$$\Delta p = \frac{-\left(-12.78\left(\frac{kJ}{mol}\right)\right)}{96.5\left(\frac{kJ}{mol}\right)}$$

$$\Delta p = 0.132V$$

With this information we now have two ways of calculating the protons required to facilitate electron transfer. First when electrons enter and leave the on the same side of the membrane we can use the simple relationship between proton motive force and the redox span:

$$n\Delta p = 2\Delta E_h$$

Where Δp is the proton motive force in mv and n is the number of protons required for translocation and the 2 for the 2 electrons transfer in the redox span. Solving for n gives:

$$n = \left(\frac{e^-\Delta E_h}{\Delta p}\right)$$

$$n = \left(\frac{2e^- * -0.196V}{0.132V}\right)$$

$$n = -2.96H^+$$

The next way to calculate using the ΔG and $\Delta\mu_{H^+}$ by understanding that in order to make the reaction favorable $\Delta G^{tot} \leq 0$

Meaning the ΔG of the redox reaction must at least equal the $\Delta\mu_{H^+}$

$$\Delta G = n \cdot \Delta\mu_{H^+}$$

or

$$\frac{\Delta G}{\Delta\mu_{H^+}} = n$$

$$\frac{\left(37.82\frac{kJ}{mol}\right)}{\left(-12.78\frac{kJ}{mol}\right)} = n$$

$$n = -2.95H^+$$

Both of ways are very similar and probably only different due rounding errors. We can round the stoichiometry to $3\ H^+/2e^-$

This is similar to the $2\ H^+/2e^-$ stoichiometery given for proton pumping with acetogens and which is annotated in most databases for RNF.

Rnf and the relationship to proton motive force

Now we can take this information and show how Rnf would be influenced by the proton motive force and how this could make it a conditionally enzyme with use under higher proton motive forces.

We can simply plot the relationship between the ΔG of Rnf and pmf and plot the linear relationship:

$$\Delta G = \Delta G^o + n * \Delta\mu_{H^+}$$

To convert to mV we will use

$$\Delta G^o = -2e^- * 0.0964\frac{kJ}{mVmol} * -196\ mV$$

$$\Delta G^o = 37.82\ kJ/mol$$

$$\Delta\mu_{H^+} = 3H^+ * 0.0964\frac{kJ}{mVmol} * -132\ mV$$

$$\Delta\mu_{H^+} = -38.2\ kJ/mol$$

To graph below we will use

$$\Delta G^{Rnf} = \Delta G^o + n^{H^+} * -pmf * F$$

Where ΔG^{Rnf} the dependent variable is the gibss free energy of the Rnf reaction is equal to the ΔG^o which is equal to $37.86\frac{kJ}{mol}$ plus independent variable pmf in mV and the number of protons translocated n^{H^+} multiplied by the Faraday constant $F = 0.0964\frac{kJ}{mVmol}$

In [2]:

```
F= .096485
def dG_pmf_rxn(d_Ev, n, d_p):
    return  d_Ev + (n*-d_p*F)

xvals = np.linspace(0,300,100 )
d_Gi_6h = dG_pmf_rxn(37.82, 2, xvals)
d_Gi_5h = dG_pmf_rxn(37.82, 3, xvals)
d_Gi_4h = dG_pmf_rxn(37.82, 4, xvals)


fig = plt.figure()
ax = plt.gca()
plt.ylim((-80,60))
plt.xlim((0,300))
ax.plot(xvals, d_Gi_6h, label =r"$\mathrm{2\ H^+/\,2e^-}$",  color = 'tab:blue')
ax.plot(xvals, d_Gi_5h, label =r"$\mathrm{3\ H^+/\,2e^-}$", color = 'tab:orange')
ax.plot(xvals, d_Gi_4h, label =r"$\mathrm{4\ H^+/\,2e^-}$", color = 'tab:green')
ax.axhline(y=0, color="black", linestyle = ":")
ax.axhline(y=-36.7, color="purple", linestyle = ":")
plt.legend(loc ='upper right')
plt.xlabel(r'$pmf _{\mathrm{(mV)}}$', fontsize = 14)
plt.ylabel(r'$\mathrm{\Delta G^{Rnf}\ _{\left(kJ \cdot mol^{-1}\right)}}$', fontsize = 14)
plt.annotate(r'$\mathrm{\Delta G^{Fix}}$', (40,-33) , fontsize = 14, color = "purple")
fig.tight_layout()
plt.savefig("./thermo_dp.tiff", dpi= 300, format="tiff")
```

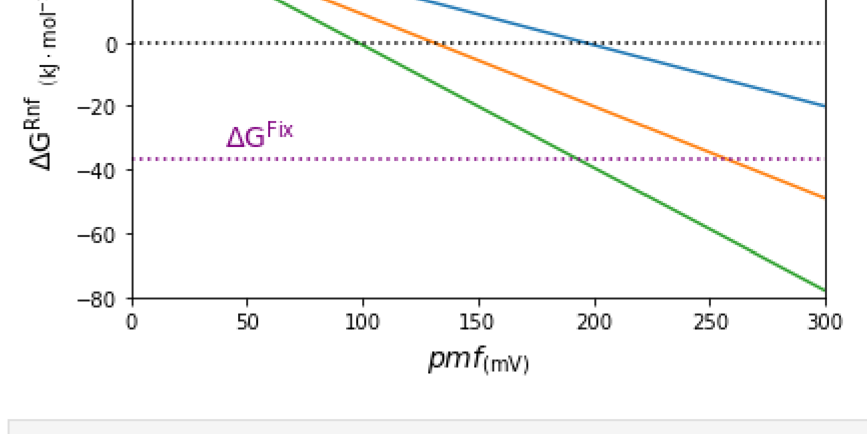

In [ ]:
